# Identification of novel fatty acid-binding protein 4 polymorphisms and association of g.5002C>T with longissimus dorsi thickness in Indonesian Bali cattle (*Bos javanicus*)

**DOI:** 10.14202/vetworld.2026.760-770

**Published:** 2026-02-26

**Authors:** Dairoh Dairoh, Mokhamad Fakhrul Ulum, Sutikno Sutikno, Ahmad Furqon, Isyana Khaerunnisa, Cece Sumantri, Jakaria Jakaria

**Affiliations:** 1 Research Center for Applied Zoology, National Research and Innovation Agency (BRIN), Bogor, 16911, West Java, Indonesia; 2 Division of Reproduction and Obstetrics, School of Veterinary Medicine and Biomedical Sciences, IPB University, Bogor, 16680, West Java, Indonesia; 3 Department of Animal Production and Technology, Faculty of Animal Science, IPB University, Bogor, 16680, Indonesia

**Keywords:** Bali cattle, *Bos javanicus*, *FABP4* gene, fatty acid composition, meat quality traits, single-nucleotide polymorphism, ultrasound imaging

## Abstract

**Background and Aim:**

The fatty acid-binding protein 4 (*FABP4*) gene is a key regulator of lipid metabolism and has been linked to carcass and meat quality traits in several cattle breeds. However, genetic variation in *FABP4* and its phenotypic relevance in Bali cattle (*Bos javanicus*), an important indigenous Indonesian breed, remains poorly characterized. This study aimed to identify novel single-nucleotide polymorphisms (SNPs) in the *FABP4* gene of Bali cattle and to evaluate their associations with *in vivo* ultrasound-measured carcass traits and fatty acid composition.

**Materials and Methods:**

Blood samples were collected from 95 Bali cattle bulls sourced from a single population. Genomic DNA was extracted, and a 721-bp fragment of *FABP4* spanning intron 2 to exon 4 was amplified and sequenced using the Sanger method. SNP detection and sequence alignment were performed using standard bioinformatics tools. Carcass and meat quality traits, including longissimus dorsi thickness (LDT), backfat thickness (BFT), marbling score (MS), and intramuscular fat (IMF), were measured in live animals using ultrasound imaging. Fatty acid composition was determined from post-mortem muscle samples using gas chromatography–flame ionization detection. Associations between *FABP4* genotypes and phenotypic traits were analyzed using a general linear model, with statistical significance set at p < 0.05.

**Results:**

Four novel *FABP4* SNPs were identified: g.4631T>C, g.4724T>C, and g.4769G>A located in intron 3, and g.5002C>T located in exon 4. The g.5002C>T variant was a nonsynonymous substitution resulting in a valine-to-alanine amino acid change. Among the identified polymorphisms, only g.5002C>T showed a significant association with LDT (p = 0.0296). Animals carrying the CT genotype exhibited greater LDT compared with CC and TT genotypes. No significant associations were observed between any *FABP4* SNPs and BFT, MS, IMF, or fatty acid composition.

**Conclusion:**

This study provides the first evidence of *FABP4* genetic variation in Bali cattle and demonstrates that the g.5002C>T polymorphism is significantly associated with LDT. The CT genotype at this locus may serve as a potential genetic marker for improving muscle development in Bali cattle, subject to validation in larger and independent populations.

## INTRODUCTION

Meat quality and carcass traits are economically important in beef production because they directly affect processing efficiency, market value, and consumer acceptance [1]. Key attributes, including muscle development, tenderness, intramuscular fat (IMF), and fatty acid (FA) composition [2], play central roles in determining meat palatability and nutritional quality. Improvements in these traits can substantially enhance the market appeal of meat products by meeting consumer expectations for superior taste and texture while also providing health benefits through improved FA profiles [3]. Inoue *et al*. [4] further reported that additional meat quality traits, particularly tenderness, contribute to unit meat price alongside the marbling score (MS).

In Indonesia, Bali cattle (*Bos javanicus*) represent an important indigenous breed and a valuable animal genetic resource domesticated from Banteng (*Bibos banteng*) [5]. Bali cattle are well known for their adaptability to tropical environments, tolerance to limited feed resources, and significant contribution to national beef production. Compared with commercial breeds, *B. javanicus* exhibits superior meat quality, with carcass yields ranging from 54.07% to 55.61% [6]. These cattle are characterized by desirable meat texture and are highly preferred in the marketplace. The meat of *B. javanicus* is reported to be tender [7], and previous studies have demonstrated favorable meat quality with a healthy FA composition, including omega-3 and omega-6 fatty acids [8].

Meat quality and carcass traits are complex phenotypes regulated by multiple genes. The application of DNA markers in marker-assisted selection (MAS) has markedly advanced livestock breeding [9]. Developments in molecular genetics have enabled the identification of candidate genes associated with economically important traits, thereby improving breeding efficiency through MAS [10, 11]. This approach allows researchers to detect specific genetic variations linked to desirable phenotypes. However, integrative genotype–phenotype analyses combining *in vivo* ultrasound measurements and FA profiling have not previously been applied to *B. javanicus*.

The fatty acid-binding protein 4 (*FABP4*) gene plays a crucial role in lipid metabolism by promoting lipid hydrolysis and the intracellular transport of FAs among tissues [12]. In cattle, *FABP4* is located on chromosome 14 and consists of four exons and three introns, encoding a protein that binds and transports FAs within cells [13]. *FABP4* facilitates FA uptake, transport, and storage in adipocytes and regulates fat deposition through interactions with lipid signaling pathways, including *PPARγ* [14], thereby influencing fat accumulation and meat quality [15]. Recent studies have reported associations between single-nucleotide polymorphisms (SNPs) in *FABP4* and marbling traits in Caracu × Nellore cows, as well as in Caracu, Canchim, and Angus sires [16], and with FA composition in Angus, Brahman, Creole, Hereford, Holstein, Limousine, and Nellore cattle [17, 18]. Nishimura *et al*. [19] demonstrated a significant association between IMF deposition and meat tenderness, underscoring the importance of *FABP4* in determining meat quality. Through its effects on lipid hydrolysis and intracellular FA transport, *FABP4* supports adipocyte development and differentiation [20], promoting IMF accumulation that enhances tenderness by altering muscle fiber structure and increasing juiciness and flavor [21–23]. Furthermore, Bayraktar *et al*. [24] evaluated the expression of *FABP3* and *FABP4* in a native Indonesian cattle breed, highlighting their important roles in FA metabolism and local breed adaptation.

Despite growing evidence that *FABP4* polymorphisms are associated with carcass traits, IMF deposition, and FA composition in several taurine and crossbred cattle populations, comparable information for Bali cattle remains very limited. In particular, the genetic diversity of *FABP4* in Bali cattle and its relationship with carcass and meat quality traits assessed *in vivo* has not been clearly established. Most existing studies have examined either molecular variation or phenotypic traits in isolation, while integrated genotype–phenotype approaches combining *in vivo* ultrasonography and FA profiling are still lacking in Bali cattle. This gap constrains the identification of robust genetic markers that could be implemented in MAS programs to improve meat quality in this indigenous breed.

Therefore, this study aimed to identify and characterize SNPs in the *FABP4* gene of Bali cattle and to evaluate their associations with *in vivo* ultrasound-measured carcass traits, including longissimus dorsi thickness (LDT), backfat thickness (BFT), MS, IMF, and FA composition. By integrating molecular genetic information with phenotypic measurements, the study sought to assess the potential of *FABP4* polymorphisms as candidate markers for MAS and to provide foundational data to support genetic improvement and conservation strategies in Bali cattle.

## MATERIALS AND METHODS

### Ethical approval

The Animal Ethics Committee of the Banjarmasin City Food Security, Agriculture, and Fisheries Service approved the experimental procedures (approval number: 520/624/DKP3/X11/2021). All sampling procedures complied with established animal welfare guidelines. Gentle restraint, appropriately sized needles, and rapid venipuncture performed by trained personnel were used to minimize pain and stress during blood collection. The puncture site was cleaned before and after sampling to reduce discomfort and promote healing. Slaughter was conducted at a licensed slaughterhouse in accordance with Indonesian regulations for humane handling and slaughter of cattle.

### Study period and location

The experiment was conducted from December 2022 to June 2023. A total of 95 Bali cattle bulls from a single population were included, and all available animals were used as representative samples. The animals weighed 250–350 kg and were 18–36 months of age. Bali cattle were sourced from Kupang, East Nusa Tenggara Province, Indonesia, and transported by ship to the Basirih slaughterhouse in Banjarmasin. Upon arrival, cattle were maintained under an intensive management system for 2 weeks before slaughter in South Banjarmasin, South Kalimantan. During this period, forage was provided at 10% of body weight, concentrate at 2%, and water *ad libitum*. Animals were restrained using a cattle chute to minimize movement during blood collection by a trained veterinarian.

### Blood collection and DNA extraction

Blood samples were collected from the jugular vein using a Venoject with a sterile disposable 21-gauge needle (Becton, Dickinson and Company, Franklin Lakes, NJ, USA). Approximately 5 mL of blood was transferred into vacuum tubes containing 1.5 mL ethylenediaminetetraacetic acid (EDTA). Samples were immediately placed on ice and stored at 4°C until DNA extraction. Genomic DNA was extracted using the Geneaid Genomic DNA Mini Kit (Geneaid Biotech Ltd., Taipei, Taiwan) according to the manufacturer’s instructions. Extracted DNA was stored at −20°C. DNA purity was assessed spectrophotometrically, with acceptable A260/280 ratios of 1.8–2.2 and concentrations ≥20 ng/μL.

### Polymerase chain reaction (PCR) amplification of *FABP4*

Primer sequences were designed using Primer3 and BLAST primer tools. The forward (5′-CCC TCC ATC ATT GTA ATC ACT-3′) and reverse (5′-GGA CAA CGT ATC CAG CAG AAA-3′) primers for the *FABP4* gene amplified a fragment spanning intron 2 to exon 4. The expected PCR product length was 721 base pairs (bp). Amplification was performed using an AB System thermocycler (Applied Biosystems, Foster City, CA, USA) under the following conditions: pre-denaturation at 95°C for 1 min, followed by 35 cycles of denaturation at 95 °C for 15 s, annealing at 57°C for 15 s, extension at 72°C for 10 s, and a final extension at 72°C for 3 min.

### Agarose gel electrophoresis and DNA sequencing

PCR products were separated on a 1% agarose gel stained with Florosafe (1^st^ BASE, Singapore, Singapore) and visualized using a UV transilluminator (Bio-Rad, Hercules, CA, USA). Sequencing was performed at 1st BASE Laboratory Services (Selangor, Malaysia) using an ABI PRISM system with the BigDye Terminator kit v3.1 (Applied Biosystems). Sequencing quality was manually examined using FinchTV, BioEdit, and Molecular Evolutionary Genetic Analysis software. The first and last 30 bp were excluded because of low peak quality, and only SNPs with clear chromatogram peaks were retained. Sequence alignment was conducted against the Ensembl *Bos taurus* RefSeq transcript ENSBTAG00000037526, and SNP positions were determined using the *Bos taurus* ARS-UCD2.0: CM008181.2 reference genome assembly.

### Ultrasound imaging measurements

**Figure 1:**
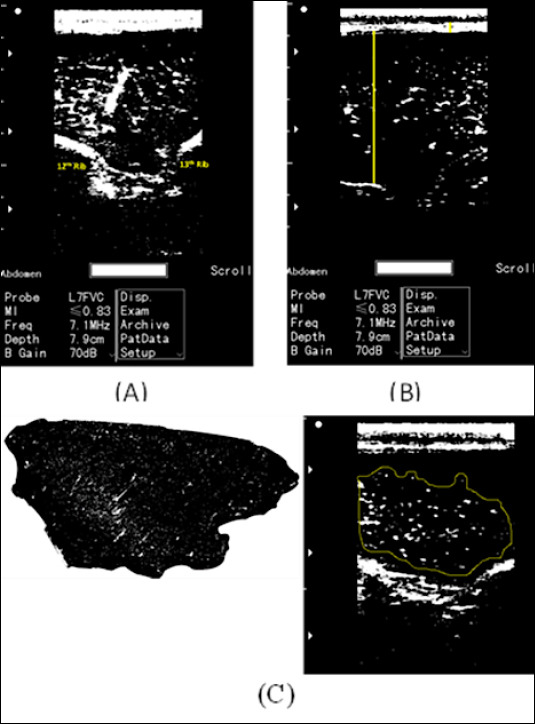
Ultrasound image analysis using ImageJ software in live Bali cattle. (A) Ribeye ultrasound image obtained between the 12th and 13th ribs. (B) Longitudinal ultrasound image used to measure backfat thickness and *longissimus dorsi* muscle thickness. (C) A transverse ultrasound image was used to estimate the percentage of intramuscular fat.

*In vivo* carcass and meat traits were measured using a 7.1-MHz ultrasound transducer applied to the left rib area. A portable ultrasound device (SIUI CTS-800, SIUI Medical Systems, Shantou, China) was used to measure LDT, BFT, MS, and IMF at the 12th–13th rib position (Figure 1A) using transverse and longitudinal scans [25, 26]. IMF sonograms were obtained 3 days before slaughter. Hair at the scanning site was shaved, and ultrasound gel was applied to ensure adequate transmission of sound waves. Images were displayed in real time, saved, and analyzed using ImageJ software (National Institutes of Health, Bethesda, MD, USA). ImageJ was used to calibrate scale units to millimeters. Measurements of BFT, LDT (Figure 1B), and IMF (Figure 1C) were recorded at three points per animal. IMF values were subjected to simple linear regression to estimate the IMF percentage. Marbling was scored according to AUS-MEAT and MSA standards, with scores ranging from 0 to 9 (AUS-MEAT, 2018).

### FA methyl ester preparation

Approximately 250 g of tenderloin muscle was collected from the right side of each carcass about 1 h post-mortem and stored at −20°C. From this sample, 40 g was used for FA analysis. Lipids were extracted using a chloroform–methanol solution, followed by transesterification to produce fatty acid methyl esters (FAMEs). FAMEs were extracted, centrifuged, dried in hexane, resuspended in chloroform to remove impurities, and purified by solid-phase extraction.

### Gas chromatography–flame ionization detector conditions

FAMEs (1 μL) were analyzed using gas chromatography equipped with a flame ionization detector. Separation was achieved on a cyanopropyl methyl sil capillary column (60 m × 0.25 mm, 0.25 μm film thickness) with nitrogen as the carrier gas at 30 mL/min. Injector and detector temperatures were set at 220°C and 240°C, respectively. The oven temperature program was 125°C for 5 min, then increased to 225°C at 10, 5, and 3°C/min, with holding times of 5, 10, and 7 min, respectively. The split ratio was 1:80, the injection volume was 1 μL, and the linear velocity was 23.6 cm/s. FAMEs were quantified as relative percentages using a mixed FAME standard for retention-time and peak-area comparisons. Quality control was ensured by standard injections before sample analysis. Fatty acids were grouped into saturated fatty acids (SFA), monounsaturated fatty acids (MUFA), and polyunsaturated fatty acids (PUFA) [27]. Total unsaturated fatty acids (UFA) were calculated as the sum of MUFA and PUFA. Analyses were performed in duplicate, and instruments were calibrated according to the manufacturer’s guidelines.

### Population genetics and SNP diversity analysis

Allele and genotype frequencies, observed and expected heterozygosity, and Hardy–Weinberg equilibrium were calculated using PopGen 1.32 software. Polymorphic information content (PIC) was calculated using the formula:


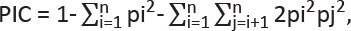


where p_i_ and p_j_ represent allele frequencies at a given locus.

### Statistical analysis

Associations between *FABP4* genotypes and carcass traits, meat quality traits, and FA composition were analyzed using a general linear model (GLM) in SAS version 9.4 (SAS Institute Inc., Cary, NC, USA). Duncan’s multiple-range test was used for post hoc comparisons. Statistical significance was set at p < 0.05. The GLM was expressed as:

Y_ij_ = μ + G_i_ + e_ij_,

where Y_ij_ is the phenotypic observation, μ is the overall mean, G_i_ is the genotype effect, and e_ij_ is the random error. Carcass and meat traits, including FA composition, were corrected to 36 months of age, body weight, and similar environmental conditions using the equation:

X_i_(corrected) = (X̅standard / X̅observation) × X_i_(observation)

where X_i_(corrected) is the corrected value, X̅standard is the standard group mean, X̅observation is the observation group mean, and X_i_(observation) is the observed value.

## RESULTS

### *FABP4* polymorphism

**Figure 2:**
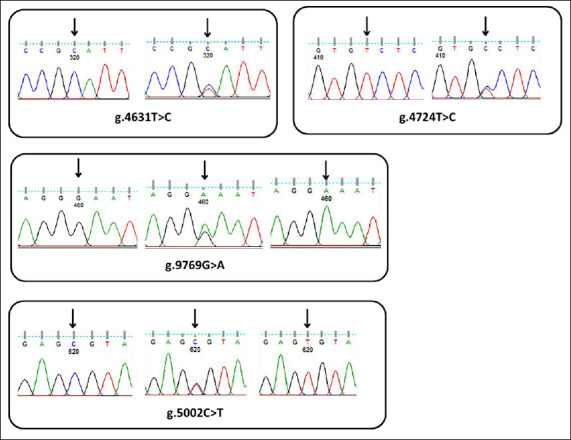
Partial sequencing maps of the *FABP4* gene in Bali cattle.

Genetic variation in *FABP4* was identified by SNP analysis of a 721 bp DNA sequencing product. SNPs were detected and characterized by examining double peaks in sequencing chromatograms using FinchTV software (Figure 2). Four novel SNPs were identified in *FABP4* from the sequencing map. Three SNPs (g.4631T>C, g.4724T>C, and g.4769G>A) were located in intron 3, whereas one SNP (g.5002C>T) was located in exon 4 (Table 1). Among these variants, g.5002C>T was a nonsynonymous substitution resulting in an amino acid change from valine to alanine, whereas the remaining three SNPs were synonymous and did not alter the amino acid sequence. Analysis of allele and genotype frequencies indicated that the AA genotype was more frequently observed for g.4724T>C, g.4769G>A, and g.5002C>T, whereas the BB genotype predominated for g.4631T>C (Table 2).

**Table 1:** SNP information of the *FABP4* gene

Gene	SNP	Location	Variation type	dbSNP	Amino acids
*FABP4*	g.4631T>C	Intron 3	Transition	Novel	–
*FABP4*	g.4724T>C	Intron 3	Transition	Novel	–
*FABP4*	g.4769G>A	Intron 3	Transition	Novel	–
*FABP4*	g.5002C>T	Exon 4	Transition	Novel	Val/Ala

Ala = Alanine, SNP = Single-nucleotide polymorphism, Val = Valine.

**Table 2:** Allelic and genotypic frequencies and diversity parameters of *FABP4* SNPs.

Gene	SNP	N	AA	AB	BB	A	B	Ho	He	χ² test	PIC
*FABP4*	g.4631T>C	95	0.00	0.08	0.92	0.04	0.96	0.084	0.081	0.160 ns	0.077
*FABP4*	g.4724T>C	95	0.93	0.07	0.00	0.96	0.04	0.074	0.071	0.119 ns	0.068
*FABP4*	g.4769G>A	95	0.78	0.21	0.01	0.88	0.12	0.211	0.206	0.051 ns	0.184
*FABP4*	g.5002C>T	95	0.84	0.14	0.02	0.91	0.09	0.137	0.164	2.721 ns	0.150

AA = Reference genotype (wild-type), AB = Heterozygous genotype, BB = Mutant genotype, *FABP4* = Fatty acid-binding protein 4 gene, He = Expected heterozygosity, Ho = Observed heterozygosity, ns = Not significant (χ² test < 3.84 at α = 0.05, df = 2), PIC = Polymorphic information content, SNP = Single-nucleotide polymorphism.

### Genetic association of *FABP4* variants with meat quality traits

The associations between *FABP4* SNPs and carcass and meat quality traits are summarized in Table 3. The SNP g.5002C>T showed a significant association with LDT (p < 0.05), suggesting a potential role in muscle development. In contrast, no significant associations were detected between *FABP4* SNPs and BFT, MS, or IMF in Bali cattle. Notably, animals carrying the CT genotype at g.5002C>T exhibited significantly greater LDT compared with those carrying the CC and TT genotypes.

**Table 3:** Association of *FABP4* SNPs with carcass ultrasound and meat characteristics in live Bali cattle.

SNP	Genotype (N)	LDT (mm)	BFT (mm)	MS	IMF (%)
g.4631T>C	TC (8)	47.24 ± 6.11	1.84 ± 0.30	1.36 ± 0.19	2.22 ± 0.47
	CC (83)	47.16 ± 5.77	1.88 ± 0.31	1.51 ± 0.56	2.58 ± 1.39
g.4724T>C	TT (84)	47.23 ± 5.76	1.89 ± 0.31	1.51 ± 0.56	2.57 ± 1.39
	TC (7)	46.51 ± 6.21	1.79 ± 0.30	1.39 ± 0.19	2.28 ± 0.48
g.4769G>A	GG (70)	46.71 ± 5.12	1.89 ± 0.32	1.51 ± 0.57	2.57 ± 1.41
	GA (20)	49.36 ± 6.99	1.84 ± 0.28	1.42 ± 0.36	2.35 ± 0.91
	AA (1)	35.17 ± nc	1.54 ± nc	2.61 ± nc	5.30 ± nc
g.5002C>T	CC (77)	46.61^b^ ± 5.73	1.86 ± 0.31	1.51 ± 0.57	2.57 ± 1.43
	CT (12)	51.18^a^ ± 4.42	2.00 ± 0.23	1.39 ± 0.23	2.28 ± 0.56
	TT (2)	44.67^ab^ ± 7.40	1.77 ± 0.57	1.76 ± 0.41	3.20 ± 1.03

BFT = Backfat thickness, *FABP4* = Fatty acid-binding protein 4 gene, IMF = Intramuscular fat, LDT = *Longissimus dorsi* thickness, MS = Marbling score, nc = Not counted. Means in the same column with different superscripts differ significantly (p < 0.05).

### Genetic association of *FABP4* variants with fatty acid composition

The relationship between *FABP4* polymorphisms and FA composition in Bali cattle is presented in Table 4. None of the identified *FABP4* SNPs showed a significant association with FA composition. Although *FABP4* polymorphisms were hypothesized to influence FA profiles, the results indicate that these variants did not contribute significantly to FA variation in Bali cattle. This suggests that other genetic factors or environmental influences may play a more dominant role in determining FA composition. Further studies are required to elucidate the interactions between *FABP4*, other candidate genes, and non-genetic factors involved in the regulation of meat quality traits in Bali cattle.

**Table 4:** Association of *FABP4* SNPs with FA composition in Bali cattle.

Fatty acid (%)	g.4631T>C TC (1)	g.4631T>C CC (43)	g.4724T>C TT (43)	g.4724T>C TC (1)	g.4769G>A GG (35)	g.4769G>A GA (9)	g.5002C>T CC (40)	g.5002C>T CT (4)
Fat content	3.57 ± nc	3.18 ± 1.23	3.18 ± 1.23	3.57 ± nc	3.17 ± 1.22	3.26 ± 1.27	3.26 ± 1.22	2.43 ± 0.92
C8:0	0.00 ± nc	0.06 ± 0.20	0.06 ± 0.20	0.00 ± nc	0.06 ± 0.21	0.04 ± 0.11	0.06 ± 0.20	0.07 ± 0.14
C12:0	0.18 ± nc	0.07 ± 0.02	0.07 ± 0.02	0.18 ± nc	0.07 ± 0.03	0.07 ± 0.01	0.07 ± 0.03	0.06 ± 0.02
C13:0	0.04 ± nc	0.03 ± 0.02	0.03 ± 0.02	0.04 ± nc	0.03 ± 0.02	0.03 ± 0.01	0.03 ± 0.02	0.03 ± 0.03
C14:0	3.35 ± nc	2.17 ± 0.53	2.17 ± 0.53	3.35 ± nc	2.15 ± 0.55	2.37 ± 0.56	2.21 ± 0.58	2.00 ± 0.27
C14:1	0.09 ± nc	0.28 ± 0.37	0.28 ± 0.37	0.09 ± nc	0.28 ± 0.38	0.26 ± 0.31	0.29 ± 0.38	0.18 ± 0.29
C15:0	0.81 ± nc	0.59 ± 0.36	0.59 ± 0.36	0.81 ± nc	0.61 ± 0.37	0.55 ± 0.26	0.60 ± 0.37	0.52 ± 0.07
C16:0	32.95 ± nc	20.82 ± 3.61	20.82 ± 3.61	32.95 ± nc	21.10 ± 4.09	21.05 ± 3.89	21.16 ± 4.17	20.43 ± 1.69
C16:1	2.00 ± nc	1.30 ± 0.35	1.30 ± 0.35	2.00 ± nc	1.31 ± 0.38	1.33 ± 0.31	1.33 ± 0.37	1.19 ± 0.12
C17:0	2.13 ± nc	1.99 ± 0.77	1.99 ± 0.77	2.13 ± nc	2.01 ± 0.83	1.92 ± 0.42	2.00 ± 0.76	1.96 ± 0.84
C17:1	0.44 ± nc	0.26 ± 0.14	0.26 ± 0.14	0.44 ± nc	0.27 ± 0.14	0.26 ± 0.16	0.27 ± 0.14	0.22 ± 0.17
C18:0	36.55 ± nc	31.91 ± 3.64	31.91 ± 3.64	36.55 ± nc	32.00 ± 4.01	32.09 ± 2.01	32.11 ± 3.65	31.05 ± 4.32
C18:1n9c	0.10 ± nc	12.45 ± 4.51	12.45 ± 4.51	0.10 ± nc	11.64 ± 5.00	14.21 ± 3.65	12.06 ± 5.04	13.25 ± 1.70
C18:1n9t	1.72 ± nc	3.03 ± 1.25	3.03 ± 1.25	1.72 ± nc	2.86 ± 1.25	3.56 ± 1.17	3.07 ± 1.28	2.27 ± 0.71
C18:2n6c	3.88 ± nc	1.75 ± 0.76	1.75 ± 0.76	3.88 ± nc	1.83 ± 0.91	1.67 ± 0.23	1.75 ± 0.78	2.27 ± 1.18
C18:3n3	0.00 ± nc	0.23 ± 0.27	0.23 ± 0.27	0.00 ± nc	0.21 ± 0.27	0.28 ± 0.26	0.21 ± 0.26	0.41 ± 0.29
SFA	77.19 ± nc	58.56 ± 5.99	58.56 ± 5.99	77.19 ± nc	58.98 ± 7.05	59.01 ± 4.42	59.16 ± 6.60	57.16 ± 6.66
MUFA	10.40 ± nc	20.31 ± 4.32	20.31 ± 4.32	10.40 ± nc	19.47 ± 4.50	22.48 ± 4.02	20.04 ± 4.68	20.61 ± 2.90
PUFA	5.14 ± nc	17.65 ± 4.75	17.65 ± 4.75	5.14 ± nc	16.71 ± 5.12	19.91 ± 4.11	17.38 ± 5.27	17.27 ± 2.30
UFA	5.26 ± nc	2.66 ± 1.09	2.66 ± 1.09	5.26 ± nc	2.76 ± 1.26	2.57 ± 0.51	2.66 ± 1.12	3.34 ± 1.34
Total FA	87.60 ± nc	78.66 ± 7.03	78.66 ± 7.03	87.60 ± nc	78.33 ± 7.20	80.96 ± 6.51	79.01 ± 7.10	77.44 ± 7.67

*FABP4* = Fatty acid-binding protein 4 gene, FA = Fatty acid, MUFA = Monounsaturated fatty acid, nc = Not counted, PUFA = Polyunsaturated fatty acid, SFA = Saturated fatty acid, UFA = Unsaturated fatty acid. Means in the same row with different superscripts differ significantly (p < 0.05).

## DISCUSSION

### Genetic diversity and population structure

Genetic diversity is a key determinant of adaptability and long-term sustainability in livestock populations. Based on allele and genotype frequency data, the AA genotype was more frequently observed for SNPs g.4724T>C, g.4769G>A, and g.5002C>T, whereas the BB genotype predominated for g.4631T>C (Table 2). Adequate genetic variation is essential for breeding programs aimed at improving performance and ensuring food security [28, 29]. Preservation of genetic diversity reduces the risks associated with inbreeding and genetic uniformity, thereby maintaining resilience, fitness, and protection against inbreeding depression [30].

All SNPs identified in this study were polymorphic, with major allele frequencies <99% and minor allele frequencies >1% [31]. The observed heterozygosity (Ho) was 50% for all SNPs, indicating relatively low heterozygosity in the Bali cattle population. This pattern may reflect inbreeding, genetic drift, and a small effective population size, which can increase homozygosity and allele fixation [32]. Reduced genetic variation may negatively affect overall health and adaptability, increasing susceptibility to diseases and environmental challenges [30]. All SNPs conformed to Hardy–Weinberg equilibrium, suggesting stable allele and genotype frequencies that were not significantly influenced by selection, migration, mutation, or genetic drift [33]. This equilibrium indicates population stability and limited disturbance from breeding practices or environmental pressures [34]. In addition, all SNPs in *FABP4* exhibited low PIC values, consistent with the limited genetic variability commonly reported in indigenous breeds with narrow genetic bases [35, 36].

### Association of *FABP4* polymorphisms with meat quality traits

The *FABP4* gene has been widely investigated for its role in regulating meat quality traits in cattle, particularly those related to lipid metabolism and muscle development. Genes involved in these pathways influence key traits such as IMF deposition and muscle thickness. In the present study, SNP g.5002C>T showed a significant association with LDT (p < 0.05), indicating its potential involvement in muscle development. This nonsynonymous substitution results in an amino acid change that may alter *FABP4* protein function, supporting its candidacy as a genetic marker for selective breeding in Bali cattle.

Previous studies have reported significant associations between *FABP4* SNPs and MS and BFT, particularly for variants located in exon 3 [37]. Gao *et al*. [38] observed associations between *FABP4* diversity and LDT as well as ultrasonographic IMF in Qinchuan cattle. Associations with hump height and eye muscle area have also been reported in northern Australian crossbred cattle [39], while Shin *et al*. [40] demonstrated relationships between *FABP4* variants and MS in Hanwoo cattle. In contrast, the lack of association between SNPs and MS, IMF, and BFT in the present study may be attributed to unbalanced genotype distributions. For example, only one AA genotype was observed for SNP g.4769G>A, precluding robust association analysis. Differences in population structure, sample size, and genetic background across studies can lead to variable allele and genotype frequencies and influence statistical outcomes [41].

### Association of *FABP4* polymorphisms with fatty acid composition

The absence of significant associations between *FABP4* polymorphisms and FA composition in Bali cattle contrasts with findings reported by Bayraktar *et al*. [24], who demonstrated strong associations between *FABP4* variants and milk composition and FA profiles in Native Southern Yellow cattle. These observations highlight tissue- and breed-specific functions of the *FABP* gene family, suggesting that the influence of *FABP4* on lipid metabolism may be more pronounced in milk than in muscle or adipose tissues of Bali cattle.

Other studies have reported significant relationships between *FABP4* variants and specific FAs. Hoashi *et al*. [42] identified associations between *FABP4* polymorphisms and palmitoleic acid (C16:1) and linoleic acid (C18:2n6c) in Qinchuan cattle. Palmitoleic acid has been linked to improved metabolic health, including reduced insulin resistance [43, 44], while linoleic acid is known for its cardiovascular benefits [45, 46]. Yin *et al*. [47] identified six *FABP4* SNPs associated with fat content and MS in Yellow Yanbian cattle, and Oh *et al*. [48] reported associations with oleic acid (C18:1n9c) and monounsaturated FA content. The lack of association observed in Bali cattle may reflect the polygenic nature of FA composition, limited genetic diversity within the population, and strong environmental influences such as diet, management system, and physiological status, which can mask genetic effects [49, 50].

### Implications for marker-assisted selection

The SNP g.5002C>T in *FABP4* represents a promising candidate for MAS in Bali cattle. However, validation in larger and geographically diverse populations under varying environmental conditions is required before practical implementation. Following validation, incorporation of this SNP into national breeding programs could facilitate the identification of animals with favorable genotypes, improve meat quality efficiency, and accelerate genetic improvement in Bali cattle [51].

## CONCLUSION

This study identified four novel SNPs in the *FABP4* gene of Bali cattle, three located in intron 3 and one nonsynonymous variant (g.5002C>T) in exon 4. Among the detected variants, only g.5002C>T showed a significant association with LDT (p < 0.05), with the CT genotype exhibiting superior muscle thickness compared with CC and TT genotypes. No significant associations were observed between *FABP4* SNPs and BFT, MS, IMF, or FA composition, indicating that the genetic effect of *FABP4* in Bali cattle is trait-specific and primarily related to muscle development rather than fat deposition or FA profiles.

The significant association between *FABP4* g.5002C>T and LDT highlights its potential utility as a genetic marker for improving muscle-related carcass traits in Bali cattle. Incorporation of this SNP into MAS schemes could support more efficient selection strategies aimed at enhancing meat yield without adversely affecting other quality traits. This is particularly relevant for breeding programs focused on indigenous cattle improvement under tropical production systems.

A major strength of this work lies in the integration of molecular genetics with *in vivo* phenotyping, combining SNP discovery, ultrasound-based carcass measurements, and FA profiling. This comprehensive genotype–phenotype approach provides a robust framework for evaluating candidate genes in indigenous cattle populations. Additionally, the identification of previously unreported *FABP4* variants expands the genomic resource base for Bali cattle.

Despite these strengths, the study was conducted on a single population with a relatively limited sample size, which may have constrained the detection of associations for traits with low genetic variance or unbalanced genotype frequencies. The low heterozygosity observed for several SNPs may also have reduced statistical power, particularly for traits such as MS, IMF, and FA composition that are strongly influenced by multiple genes and environmental factors.

Future studies should validate the effect of *FABP4* g.5002C>T in larger and geographically diverse Bali cattle populations and across different management and feeding systems. Integration of this marker with additional candidate genes and genomic tools could further enhance MAS accuracy. Functional studies examining the biological impact of the g.5002C>T amino acid substitution on *FABP4* activity would also help clarify the mechanistic basis of its association with LDT.

In conclusion, this study provides the first evidence that *FABP4* genetic variation contributes to muscle development in Bali cattle, with g.5002C>T emerging as a promising marker associated with LDT. While *FABP4* polymorphisms were not linked to fat-related traits or FA composition, the findings underscore the importance of breed-specific genetic effects and support the targeted use of *FABP4* in future genetic improvement and conservation programs for Bali cattle.

## DATA AVAILABILITY

All the generated data are included in the manuscript.

## AUTHORS’ CONTRIBUTIONS

DD and JJ: Conceptualization of the study, investigation, methodology, data analysis, laboratory work, interpretation of data, and drafted, reviewed, and edited the manuscript. DD, MFU, and JJ: Collected the animal sample and performed ultrasound imaging. SS, AF, and IK: Validation, investigation, and reviewed and edit the manuscript. CS: Data analysis and revised the manuscript. All authors have read and approved the final version of the manuscript.
